# Molecular docking analysis of mefluhybenamine with lung cancer targets

**DOI:** 10.6026/973206300181186

**Published:** 2022-12-31

**Authors:** Youssef S Alghamdi

**Affiliations:** 1 Department of Biology, Turabah University College, Taif University, P.O.BOX 11099, Taif 21944, Kingdom of Saudi Arabia

**Keywords:** Lung cancer, mefluhybenamine, molecular docking, molecular dynamics simulation, nsulin-like growth factor-1

## Abstract

Lung cancer is the most prevalent type of cancer worldwide, with 2.21 million cases and 1.80 million fatalities in 2020. The main factor influencing lung cancer is smoking, and the most common form of lung cancer, non-small cell lung cancer (NSCLC), accounts for around 80% of instances compared to small cell carcinoma, and about 75% of patients are already in an advanced stage when they are detected. Despite significant early detection and therapy improvements, the five-year survival rate for NSCLC is not encouraging. Therefore, it is essential to look into the molecular origins of non-small cell lung cancer to develop more effective therapeutic strategies-the binding affinities and energy landscape with the proteins. Cyclin Dependent kinase 2 (CDK2) and Insulin-like Growth Factor 1 Receptor (IGF1) were more substantial and sustained in lung cancer that was chosen as the two primary target proteins in this. We screened the entire Drug Bank-prepared library of 1,55,888 compounds and found (2R,3R)-7-(Methylsulfonyl)-3-(2,4,5-trifluorophenyl) -1,2,3,4-tetrahydropyrido[1,2-a] benzimidazol-2-aminium (Mefluhybenamine) to be a significant inhibitor. Mefluhybenamine showed strong hydrogen bonding and other bonding topologies, such as van der Waals force, in its high docking scores of -6.168 Kcal/mol and -5.26 Kcal/mol, and ADMET results showed excellent bioavailability, remarkable solubility, no side effects, and toxicity. The molecular dynamicsimulation confirmed the compound's stability and interaction pattern for 100 ns in an SPC water medium with the slightest deviation and fluctuation. Data shows that Mefluhybenamine is a potential candidate. However, validation of the compound is essential.

## Background:

One of the deadliest cancer types is lung cancer, partly because the disease often manifests at an advanced stage and because a significant portion of its growth can occur without any symptoms [1, 2]. Lung cancer that starts there is referred to as primary lung cancer, while secondary lung cancer is a type of cancer that develops elsewhere in the body and then spreads to the lungs. Primary lung cancer comes in two basic types [3, 4]. These are categorised according to the sort of cells that cancer first appears in,as follows:

 Non-small cell lung cancer- More than 87 percent of lung cancer occurrences are non-small-cell lung cancer, making it the most prevalent type. The three possible forms are squamous cell carcinoma, adenocarcinoma, and large-cell carcinoma [5, 6].

Small cell lung cancer- A less frequent type of lung cancer called small-cell lung cancer typically spreads more quickly than non-small-cell lung cancer [7, 8].

Lung cancer indicators are not commonly available in the advanced stages. Early symptoms include expected back discomfort and warning indications like shortness of breath. Back discomfort might develop when tumours impinge on the patient's lungs, spread to the spinal cord, or affect the ribs. Lung cancer's early signs can be challenging to detect, but the earlier it shows some diagnosis features. However, a better chance of treatment options is available if it is identified in its earlier stage [8,9, 10, 11,12]. Lung cancer risk factors include a family history of the disease. Comparing people who do not have a family with lung cancer to those who do, there may be a twofold increase in risk. Because lung cancer typically runs in families and family members are exposed to indirect smoke,it can be difficult to distinguish between an enhanced risk of the disease brought on by a family's ancestors and exposure to cigarette smoke [1, 4, 5]. Some people with early lung cancer experience symptoms, even for the most part, the symptoms do not appear until cancer has spread. The most typical symptoms of lung cancer are a persistent or worsening cough, chest pain that frequently worsens with coughing, laughter or deep breathing, hoarseness, decreased appetite, unexplained weight loss, breathing difficulty,feeling exhausted or fragile, recurrent or persistent infections like bronchitis and pneumonia, and newly appearing wheezing. Numerous academic works have suggested that multitargeted therapy may be preferable. However, the side effects still need to be assessed for accurate interpretation [13,14,15, 16,17,18]. During this study, we identified the compound name (2R,3R)-7-(Methylsulfonyl)-3-(2,4,5-trifluorophenyl)-1,2,3,4-tetrahydropyrido [1,2-a]benzimidazol-2-aminium (Mefluhybenamine) that shows good docking score and stability against Lung Cancer. Also, we performed Molecular Mechanics/Generalized Born Surface Area (MM/GBSA) calculations and screening through various computing methods like High Throughput Virtual Screening (HTVS), Standard Precision (SP), and Extra Precise (XP) [19, 20,21,22, 23,24]. Further, the study was extended to the molecular dynamics simulation for 100ns and examined various outputs.

##  Methods:

## Protein Preparation:

We considered two proteins, Cyclin-Dependent kinase 2 (CDK2) with PDBID: 1AQ1 and Insulin-like Growth Factor 1 Receptor (IGF1) with PDBID: 1K3A, which show primary indicators and play a role in the pathogenesis of lung cancer [25, 26].Protein crystal structure (3D structure) was taken from the RCSB database and imported into the Schrodinger Maestro workspace for preparation and optimisation.In 1AQ1, there is chain A with solvent and ligands; however, we kept chain A as it is and removed solvent from the protein. In the case of 1K3A, chain A and solvent were present; after preparation, we deleted the solvent and kept chain A. Both proteins were prepared with the same parameters. In the preprocess tab, assign bond orders is used by the CCD database, add hydrogens, create zero-order bonds to metals, create disulphide bonds, using Prime fill in missing side chains and missing loops, delete waters beyond 3Å from groups and generate states using Epik at pH 7 [24, 27,30]. The structure was refined by H-bond assignment with sample water orientations and optimised; we used PROPKA at pH 7 and removed waters beyond 3Å. The protein's energy was minimised that obtain the lowest state using the OPLS4 force field [31, 32].

##  Ligand Preparation and ADMET analysis:

Drug Bank is a vast online database with free access to information on drug candidates. We downloaded the complete Drug Bank library and used the LigPrep tool in maestro to prepare the complete library [24, 33]. The number of ligand sizes was kept at 500 atoms,and the OPLS4 forcefield has set at the ionisation state of Epik, which gives possible states at a target pH of 7 and gives the tautomers. The stereoisomers generated at most 32 per ligand and retained specific chiralities. Further, the QikProp tool was used to calculate the ADMET properties of the ligands kept in check during analysis to pass the ligand that satisfies the criteria [34].

##  Molecular Docking:

The prepared proteins were used for the grid generation using the Glid Grid generator tool, which was generated around the complete protein to perform the blind docking to find the optimal position for the ligand. The Virtual Screening Workflow (VSW) tool was used to do the screening. The prepared library was browsed and verified in VSW before QikProp was used to generate the ADMET attributes that had been Lipinski's Rule prefiltered [34, 35]. The docking tab was used to pick Epik state penalties, which were docked with High throughput virtual screening, standard precisiondocking, and then extra precision docking and post-process using Prime MM\GBSA [36, 37].Only the top 5% of the HTVS data were used for the SP and the top 10% for the next phase of XP docking. The binding free energy and surface were calculated for the MM\GBSA using 100% of the XP data. In order to calculate and sort the data, we exported the data into CSV format to analyse which molecule has the most potential for binding to each of the chosen protein targets. We have identified (2R,3R)-7-(Methylsulfonyl)-3-(2,4,5-trifluorophenyl)-1,2,3,4-tetrahydro pyrido[1,2-a] benzimidazol-2-aminium (Mefluhybenamine) as a drug candidate against targets through the docking process. Further, after analysing, we merged the protein-ligand to generate the complex for Molecular Dynamic Simulation.

##  Molecular Dynamics Simulation:

Biologists frequently utilise the approach known as molecular dynamics (MD) to simulate the physical stability and flexibility of atoms and molecules in order to assess the complexity and stability of any protein or protein-ligand complex in various solute mediums and temperatures. The Desmond package, considered the MD algorithm's quickest and most accurate calculation, was used to run the MD simulation [38]. The 3D boundary set-up was in an orthorhombic boundary box with 10Å x 10Å x 10Å in the buffer state, and the system in the SPC water model was constructed using the System Builder tool [39]. Additionally, the system is built for MD simulation by adding 6Cl- ions after the salt and ions placement within 20 have been removed. This neutralises the system in both circumstances. Additionally, 1000 frames are produced at NPT ensemble class with a simulation production run of 100ns and a recording interval (ps) trajectory of 1000 for each complex [40]. Each complex was kept at 1.01325 bar of pressure and 300 K in temperature. Additionally, the produced trajectory was evaluated using the simulation interaction diagram (SID) tool.

##  Results and Discussion:

## Interaction analysis and ADMET evaluation:

Computerised criteria are defined for each candidate that is analysed and categorised in the event of a docking score, and through the docking parameters, we have identified a compound name Mefluhybenamine against Lung Cancer. We have also demonstrated extensive analyse of the interactions between ligands and proteins. Mefluhybenamine with Cyclin-Dependent Protein Kinase 2 (CDK2) also PDBID: 1AQ1 has been shown to have a docking score of -6.168 Kcal/mol and MM\GBSA score of -37.7 Kcal/mol (Table 1-see PDF), and it was fit to inside of the pocket (Figure 1A-see PDF) while interacting through the hydrogen bond with LYS129 residue with O atoms. Another hydrogen bond also formed among ASP86 residue with NH3+ atom, and one salt bridge also formed among the interaction of ASP86 with NH3+ atom of the ligand (Figure 1B-see PDF). While in the case of Insulin-like Growth Factor 1 Receptor (IGF1) in complex with the ligand Mefluhybenamine (PDBID: 1K3A), has induced a docking score of -5.26 Kcal/mol and MM\GBSA score of -38.65 Kcal/mol (Table 1-see PDF) when fitted inside the binding pocket (Figure 1C-see PDF) that interacts through the hydrogen bonding formed among the THR1053 residue and NH3+ atom of the ligand (Figure 1D-see PDF). Other than the docking and MM\GBSA scores, more interaction scores such as Prime Hydrogen bonds, Prime Vander wall's forces, ligand efficiency sa and ligand efficiency are providedin Table 1(see PDF). The standard values against the descriptor are given while comparing the computed values of the ligand Mefluhybenamine with the QikProp tool for fully comprehending the ADMET. All the calculated values of the ligand satisfy all criteria and indicate that the Mefluhybenamine compound can be used against Lung Cancer (Table 2-see PDF). Although it also shows no significant side effects and toxicity, experimental validation in the in-vitro condition is essential before moving to the in-vivo or human use.

##  Molecular Dynamics Simulation analysis:

Molecular Dynamics (MD) simulation is a computation tool for analysing the molecular and atomic motions and physical properties of ligand-protein complex interactions and stability. In the present study, we considered MD simulations of complexes kept at 100ns time duration with 1000 recordings of each complex individually in the SPC water model to analyse the Root Mean Square Deviation (RMSD), Root Mean Square Fluctuation (RMSF) and Simulative Interaction Diagram (SID). Further, the detailed analysis is as follows:

##  Root-Mean-Square Deviation:

Root Mean Square Deviation (RMSD) is commonly used to calculate the statistical information that reveals the strength of the bonds between proteins and ligands. RMSD provides the molecular level deviation against time duration in a nanosecond with Angstrom value Å. The Cyclin-Dependent Protein Kinase 2 (1AQ1) protein with Mefluhybenamine has initially deviated to 1.13 Å, and the ligand shows deviation to 0.48 Å at 0.10 ns, while at 100 ns, the protein CDK2 shown deviation at 2.28 Å and Mefluhybenamine showed deviation at 26.27 Å. If the initial deviation is ignored, the complete RMSD of protein and ligand was trustable and acceptable (Figure 2A-see PDF). For the complete 100ns, protein has shown deviations below 2 Å. However, the ligand has shown deviation to some extent. The most deviation was during 40ns-60ns, and after the 60ns ligand also stabilised and showed a deviation of less than 2 Å. From the above points, it can be stated that the complex is stable, and it is suggested to take the analysis in in-vitro conditions. The Insulin-like Growth Factor 1 Receptor (1K3A) protein has an initial deviation of 1.25 Å, and ligand Mefluhybenamine provides a deviation of 1.27 Å at 0.10 ns. At 100 ns, protein deviated at 2.93 Å while ligand deviated at 7. Å. The whole complex was initially and then stabilised. However, after ignoring the initial deviation stage, the complex was satisfied (Figure 2B). The protein and ligand both deviated till 10ns and then showed stable performance. The comparative understanding suggested that the complex of Insulin-like Growth Factor-1 (1K3A) with Mefluhybenamine has shown more stable performance during simulative analysis, while the Cyclin-Dependent Protein Kinase-2 (1AQ1) with ligand Mefluhybenamine has shown deviation till some extent.

##  Root-Mean-Square Fluctuation:

Root Mean Square Fluctuation (RMSF) plot shows the protein (c-alpha) and ligand fluctuation at atomic and residue index. In the plot, the protein (c-alpha) is shown in blue, whereas the ligand is green against the residue index. The protein Cyclin-Dependent Protein Kinase-2 (1AQ1) with ligand Mefluhybenamine has achieved very steadily, not much fluctuation, and only a few residues show fluctuation that gone beyond 2 Å are LEU25, THR26, PHE152, GLY153, VAL154, PRO155, VAL156, THR158, TYR159, THR160, HIS161, GLU162, VAL164, VAL289, LEU296, ARG287, LEU298, NMA298 (Figure 3A). The Insulin-like Growth Factor-1 Receptor (1K3A) with ligand Mefluhybenamine was noticed that a few fluctuations residues are VAL958, PRO959, ASP960, GLU961, GLY976, GLN977, GLY978, SER979, PHE980, GLY981, GLY990, VAL991, VAL992, LYS993, ASP994, GLU995, PRO996, GLU997, GLU1007, ALA1008, ALA1009, SER1010, MET1011, ARG1012, GLU1013, ILE1015, GLU1016, GLY1042, PRO1044, GLU1067, MET1068, GLU1069, ASN1070, ASN1071, PRO1072, VAL1073, LEU1074, ALA1075, PRO1076, GLY1139, GLY1140, LYS1141, GLY1142, LEU1144, GLY1185, SER1187, ASN1188, LYS1256, NMA1256 (Figure 3B-see PDF). When analysing the whole RMSF plot, the protein was steady, and it gives many interactions with the ligand Mefluhybenamine, accepted that ligand Mefluhybenamine has fit and stable that entering into the pocket to stop the action and activity of the protein. The figure's green colour shows the ligand contacts during the simulative period. While comparing both conditions, Mefluhybenamine has shown many interactions with 1AQ1, dispersed sufficiently from initial to end residues.

## Simulative interaction analysis:

The SID or the Simulative Interaction Diagram proves that the interaction between ligand and protein during the simulation periods was much better with web-like formations. At the same time, to understand the bonding types and dynamic angles of residues, we have extracted the interaction count for both conditions and plotted it in figure 4. The complex of protein Cyclin-Dependent Protein Kinase-2 (1AQ1) with ligand Mefluhybenamine gives enormous interaction bonding by Pi-Pi stacking with benzene ring among PHE4 residue, and hydrogen bonds interact with ASP145, ASN23 residues with N atom, hydrogen bonds also interact with ASP86, GLN131 residues with NH3+ atom of the ligand Mefluhybenamine (Figure 5A-see PDF). The Insulin-like Growth Factor 1 Receptor (1K3A) with ligand Mefluhybenamine interacts with hydrogen bonding among ASP1056 with NH3+ atom; hydrogen bond also interacts with MET1052 with O atom of the ligand Mefluhybenamine (Figure 5B-see PDF).

## Conclusion:

Lung diseases are widespread nowadays, and lung cancer is among the most dangerous. While compared to other cancers, lung cancer remains at the top and is on the world health organisation's top priority list to find a better cure. In this study, we have screened a huge library and identified a drug compound name (2R,3R)-7-(Methylsulfonyl)-3-(2,4,5-trifluorophenyl)-1,2,3,4-tetrahydro pyrido [1,2-a]benzimidazol-2-aminium (Mefluhybenamine) that is showing an excellent binding score with two main proteins participating in the case of lung cancers are Insulin-like Growth Factor-1 and Cyclin-Dependent Protein Kinase-2. Also, the complexes have shown good MM\GBSA scores and ligand efficiency during the ADMET analysis. Further, the simulation extended the analysis for validation, and the results exceeded expectations. From the above analysis, it can be concluded that the identified drug can work for lung cancer again. However, validation is needed before any prescription.
